# Proteome analysis, genetic characterization, and antibiotic resistance patterns of *Klebsiella pneumoniae* clinical isolates

**DOI:** 10.1186/s13568-024-01710-7

**Published:** 2024-05-09

**Authors:** Eman Marzouk, Adil Abalkhail, Jamaan ALqahtani, Khalid Alsowat, Menwer Alanazi, Feras Alzaben, Abdulaziz Alnasser, Anas Alasmari, Mohammed Rawway, Abdelmaged Draz, Akram Abu-Okail, Abdulmohsen Altwijery, Ihab Moussa, Sulaiman Alsughayyir, Saleh Alamri, Mohammed Althagafi, Abdulrahman Almaliki, Ahmed Elnadif Elmanssury, Ayman Elbehiry

**Affiliations:** 1 Department of Public Health, College of Applied Medical Sciences, Qassim University, Buraydah, 51452 P.O. Box 6666, Saudi Arabia; 2 Family Medicine Department, King Fahad Armed Hospital, 23311 Jeddah, Saudi Arabia; 3 Pharmacy Department, Prince Sultan Armed Forces Hospital, 42375 Medina, Saudi Arabia; 4 Dental Department, King Salman Armed Forces Hospital, 47521 Tabuk, Saudi Arabia; 5 Department of Food Service, King Fahad Armed Forces Hospital, 23311 Jeddah, Saudi Arabia; 6 Psychiatry Department, Prince Sultan Military Medical City, 11632 Riyadh, Saudi Arabia; 7 Neurology department, king Fahad military hospital, 23311 Jeddah, Saudi Arabia; 8 Biology Department, College of Science, Jouf University, 42421 Sakaka, Saudi Arabia; 9 Botany and Microbiology Department, Faculty of Science, Al-Azhar University, 71524 Assiut, Egypt; 10 Department of Veterinary Preventive Medicine, College of Veterinary Medicine, Qassim University, 52571 Buraydah, Saudi Arabia; 11 Department of Food Since, King Saud University, Riyadh, Saudi Arabia; 12 Department of Botany and Microbiology, College of Science, King Saud University, 11451 Riyadh, Saudi Arabia; 13 Medical Administration, Armed Forces Medical Services, 12426 Riyadh, Saudi Arabia; 14 Prince Sultan Military Medical City, 13525 Riyadh, Saudi Arabia; 15 Laboratory Department, Armed Forces Center for Health Rehabilitation, 21944 Taif, Saudi Arabia; 16 Physiotherapy Department, Armed Forces Center for Health Rehabilitation, 21944 Taif, Saudi Arabia

**Keywords:** *Klebsiella pneumoniae*, Proteome identification, Genomic analysis, Antibiotic resistance, Public health

## Abstract

*Klebsiella pneumoniae* (*K. pneumoniae*) is a member of the ESKAPE group and is responsible for severe community and healthcare-associated infections. Certain *Klebsiella* species have very similar phenotypes, which presents a challenge in identifying *K. pneumoniae*. Multidrug-resistant *K. pneumoniae* is also a serious global problem that needs to be addressed. A total of 190 isolates were isolated from urine (*n* = 69), respiratory (*n* = 52), wound (*n* = 48) and blood (*n* = 21) samples collected from various hospitals in the Al-Qassim, Saudi Arabia, between March 2021 and October 2022. Our study aimed to rapidly and accurately detect *K. pneumoniae* using the Peptide Mass Fingerprinting (PMF) technique, confirmed by real-time PCR. Additionally, screening for antibiotic susceptibility and resistance was conducted. The primary methods for identifying *K. pneumoniae* isolates were culture, Gram staining, and the Vitek® 2 ID Compact system. An automated MALDI Biotyper (MBT) instrument was used for proteome identification, which was subsequently confirmed using SYBR green real-time polymerase chain reaction (real-time PCR) and microfluidic electrophoresis assays. Vitek® 2 AST-GN66 cards were utilized to evaluate the antimicrobial sensitivity of *K. pneumoniae* isolates. According to our results, Vitek® 2 Compact accurately identified 178 out of 190 (93.68%) *K. pneumoniae* isolates, while the PMF technique correctly detected 188 out of 190 (98.95%) isolates with a score value of 2.00 or higher. Principal component analysis was conducted using MBT Compass software to classify *K. pneumoniae* isolates based on their structure. Based on the analysis of the single peak intensities generated by MBT, the highest peak values were found at 3444, 5022, 5525, 6847, and 7537 m/z. *K. pneumoniae* gene testing confirmed the PMF results, with 90.53% detecting entrobactin, 70% detecting *16 S rRNA*, and 32.63% detecting ferric iron uptake. The resistance of the *K. pneumoniae* isolates to antibiotics was as follows: 64.75% for cefazolin, 62.63% for trimethoprim/sulfamethoxazole, 59.45% for ampicillin, 58.42% for cefoxitin, 57.37% for ceftriaxone, 53.68% for cefepime, 52.11% for ampicillin-sulbactam, 50.53% for ceftazidime, 52.11% for ertapenem, and 49.47% for imipenem. Based on the results of the double-disk synergy test, 93 out of 190 (48.95%) *K. pneumoniae* isolates were extended-spectrum beta-lactamase. In conclusion, PMF is a powerful analytical technique used to identify *K. pneumoniae* isolates from clinical samples based on their proteomic characteristics. *K. pneumoniae* isolates have shown increasing resistance to antibiotics from different classes, including carbapenem, which poses a significant threat to human health as these infections may become difficult to treat.

## Introduction

Among the most important and prevalent opportunistic pathogens, *Klebsiella pneumoniae* (*K. pneumoniae*) is responsible for a variety of severe community and healthcare-associated infections (HAIs) (Roscetto et al. 2021; Ndiaye et al., 2023). In recent years, *K. pneumoniae* has become increasingly multidrug-resistant, posing a serious threat to the general public (Rawy et al. 2020; Ibrahim 2023; Yang et al. 2023). Because of its propensity to develop multidrug resistance and cause deadly outcomes, this bacterium is considered a dangerous pathogen among the ESKAPE group (Bharadwaj et al. 2022; Priyamvada et al. 2022). *K. pneumoniae* can colonize the skin, oropharynx, or digestive tract of humans and cause pneumonia, soft tissue infections, wound infections, urinary tract infections, cholangitis, osteomyelitis, and meningitis, particularly in those with impaired immune systems or underlying medical conditions such as diabetes mellitus (PodschunUllmann 1998; Gierczynski et al. 2007; Remya et al. 2019).

Since *K. pneumoniae* has similar biochemical reactions to other coliforms, its isolation using conventional methods cannot be relied upon (Hansen et al. 2004; Osman et al. 2020). Healthcare settings can routinely identify a variety of clinical bacteria using automated biochemical methods such as BD Phoenix and Vitek 2 Compact systems (Dos Reis et al. 2022; Elbehiry et al. 2023). However, these techniques are inadequate for distinguishing between different bacterial species at the species level (Pesciaroli et al. 2015; Elbehiry et al. 2023). Quick identification of *K. pneumoniae* is essential for the early and targeted application of antibiotics and infection prevention (Adams-Sapper et al. 2015; Kilic et al. 2016). A delay in receiving the appropriate antibiotic therapy is a major risk factor for infection-related morbidity and mortality (Kilic et al. 2016). Therefore, the development of a method that can accurately, rapidly, inexpensively, and economically identify distinct forms of bacteria in medical settings is imperative. Recently, Peptide Mass Fingerprinting (PMF) has emerged as a powerful analytical tool for the identification and discrimination of bacteria (Elbehiry et al. 2021). It can identify and differentiate bacteria based on their unique mass spectra (Elbehiry et al. 2023). This technology is utilized in various fields, including medical research, food safety, and environmental protection (Hou et al. 2019; Abalkhail and Elbehiry 2022; Alzaben et al. 2022). Due to its ability to rapidly detect species, this technique is becoming increasingly popular as a detection approach.

Virulence factors, in conjunction with the antimicrobial susceptibility profile, may influence the spectrum of clinical infections caused by *K. pneumoniae* such as pneumonia, bloodstream infections, wound infections, and meningitis (Remya et al. 2019). To date, the capsule, lipopolysaccharides, siderophores, and fimbriae of *K. pneumoniae* have been extensively studied for potential virulence genes (Parrott et al. 2021). As a result, these factors play a critical role in adhesion, colonization, invasion, and emergence of infection. Recent discoveries include outer membrane proteins, porins, efflux pumps, iron transport systems, and genes that contribute to allantoin metabolism (Ahmed and Alaa 2016; Paczosa and Mecsas 2016). Therefore, molecular characterization of clinical isolates of *K. pneumoniae* can reveal the genes involved in infection, the mode of transmission, and the best medications to treat the illness. Antigens known as K antigens, which are components of capsules, protect bacteria from phagocytosis based on strain specificity. Of the 77 capsular serotypes associated with serious infections in humans, K1 and K2 are two of the most common (Paczosa and Mecsas 2016). K1 is the only capsule serotype in which both microviscosity-associated gene A (*magA*) and capsule-associated gene A (*K2A*) are restricted. These genes play a role in the development of liver abscesses (Seo et al. 2016; Al-Deresawi et al. 2023), and there are reports of outbreaks caused by K1 or K2 serotypes in locations other than the liver (Ku et al. 2008).

The mucoid phenotype A gene (*rmpA*) is regulated by a restriction plasmid or chromosome-mediated regulator, which regulates the production of capsular polysaccharides (Bialek-Davenet et al. 2014; De Koster 2023). It enhances capsule production in particularly virulent *K. pneumoniae*. There is growing evidence that *magA* and *k2A* genes are key in the pathophysiology of hypervirulent *K. pneumoniae* infections (Remya et al. 2019). Additionally, *K. pneumoniae* can produce enterobactin siderophores (*entB*), tiny molecules that are capable of stealing iron from the ferrous-chelating proteins of their host, enabling it to escape neutralization by the host (Willcocks et al. 2016; Kot et al. 2023; Wei et al. 2023).

Multidrug resistance has increased worldwide, posing a significant public health threat. Recent investigations have documented the emergence of multidrug-resistant bacterial pathogens from various sources, highlighting the urgent need for new, effective, and safe alternatives to antibiotics, such as probiotics (Kareem et al. 2021; Shafiq et al. 2022; El-Razik et al. 2023). Additionally, routine antimicrobial susceptibility testing is crucial for identifying the most effective antibiotic treatment, as well as screening for emerging multidrug-resistant strains (Elbehiry et al. 2024).

Currently, there is a growing concern over the emergence of antibiotic resistance among various strains of bacteria worldwide (Chinemerem Nwobodo et al. 2022; Bengtsson-Palme et al. 2023; Salam et al. 2023). The incidence of multidrug-resistant *K. pneumoniae* has increased significantly over the past few decades, representing a threat to human health (Siddique et al. 2020; Miftode et al. 2021). Due to *K. pneumoniae*’s ability to develop numerous pathways for antibiotic resistance, it is particularly dangerous as a cause of healthcare-associated infections (Elbehiry et al. 2022, 2022). Over 100 distinct antimicrobial resistance-acquired genes have been identified in *K. pneumoniae* (Moya and Maicas 2020). Many clinical isolates of *K. pneumoniae* are capable of counteracting the effects of lactam antibiotics, such as penicillins, cephalosporins, and carbapenems, which are widely used in hospitals and critical care facilities (Aminul et al. 2021). In addition to porin loss, these factors include the development of extended-spectrum beta-lactamases, ampC β-lactamases, metallo-β-lactamases, and carbapenemase (Wasfi et al. 2016). Efflux pumps are also responsible for *K. pneumoniae*’s resistance to chloramphenicol, macrolides, quinolones, and β-lactam antibiotics (Maurya et al. 2019).

To maintain a certain level of antibiotics in the blood and tissues throughout treatment, antibiotics should be administered by a physician who understands the interval-dose-response relationship (Saha 2019). The World Health Organization (WHO) reports that drug resistance can be caused by inadequate antibiotic use, transmission of resistant bacteria from patients and healthcare workers, a lack of knowledge of antibiotic application requirements, and a lack of simple evaluation methods for monitoring antibiotic limitations (Organization 2011; AlyBalkhy 2012). Additionally, a majority of the routinely recommended antibiotics, such as ampicillin, amoxicillin, cephalosporins, aminoglycosides, and -lactam antibiotics, are ineffective against *K. pneumoniae* (Jadhav et al. 2012; Cevik et al. 2022). Therefore, the current investigation aimed to utilize rapid identification technology, such as the PMF technique, along with molecular typing of virulence genes, to investigate the epidemiological spread of *K. pneumoniae* strains in healthcare settings in Al-Qassim, Saudi Arabia. Furthermore, susceptibility and resistance against *K. pneumoniae* were also evaluated for various classes of commonly used antibiotics.

## Materials and methods

### Ethical approval

Since there were no human participants in this study, there was no need for ethical approval or written authorization. We utilized only bacterial cultures obtained from routine medical tests or strain collections, rather than primary human samples. This study used clinical strains that were obtained from routine diagnostic procedures. In light of this, no attempt was made to obtain patient samples for the purpose of the inquiry.

### Bacterial isolates and preliminary identification

A total of 190 isolates were isolated from urine (*n* = 69), respiratory (*n* = 52), wound (*n* = 48) and blood (*n* = 21) samples collected from various hospitals in the Al-Qassim, Saudi Arabia, between March 2021 and October 2022. Blood agar and MacConkey agar (HiMedia Laboratories, Thane, India) were used to isolate bacteria from respiratory, wound and blood samples, while Cystine-Lactose-Electrolyte-Deficient Agar (CLED) (HiMedia Laboratories, Thane, India) was used to isolate bacteria from urine samples. Preliminary identification of *K. pneumoniae* was based on lactose-fermenting mucoid colonies on MacConkey’s and CLED agars, as well as Gram-negative rods under a light microscope after staining. Automated identification Vitek2 compact GN-card (BioMerieux, France) was also used to identify the isolates.

### Biochemical analysis of *K. pneumoniae* using automated ID instrument

The Vitek® 2 Compact (BioMérieux, Marcy l’Etoile, France) was used to identify *K. pneumoniae* by the manufacturer’s instructions. In brief, 2–3 fresh colonies were dropped into a sterilized physiological sodium chloride solution. The McFarland turbidity was adjusted to 0.50–0.63 using DensiChek™ (BioMérieux, Marcy l’Etoile, France). Then, 5 mL of the suspension was placed onto each Vitek 2 ID-GN card. Finally, the cards and suspension tubes were inserted into the Vitek 2 cassette. The unidentified bacteria were compared with the corresponding reference strains stored in the Vitek 2 Compact software to ensure precise identification.

### The identification of *K. pneumoniae* using peptide Mass Fingerprinting technique

According to the ethanol-formic acid-acetonitrile extraction protocol created by Bruker Daltonics in Bremen, Germany, the MALDI Biotyper (MBT) (Lartigue 2013) was used to identify isolates of *K. pneumoniae* from diverse clinical samples. In brief, the isolates were streaked on Trypticase Soy Agar (Hardy Diagnostics, Saudi Arabia) and incubated at 37 °C for 24 h. The MBT 96 target plate (Bruker Daltonics, Bremen, Germany) was then inoculated with two duplicates of one fresh colony from each isolate to minimize the impact of randomization. We mixed the colony with one µL of a matrix solution containing saturated α-Cyano-4-hydroxycinnamic acid (CHCA), 50% acetonitrile (MeCN), and 2.5% trifluoroacetic acid (TFA). Identification was performed on the MBT (337 nm) using the Flex software from Bruker Daltonics. At a voltage of 20 kV, ions were accelerated in the positive ion mode. The ions’ pulsed extraction process was developed for ions with a mass of 1000 Da.

Spectra were then automatically collected with Flex control of laser intensity using the Compass software and analyzed by comparing typical patterns to those of fourteen *K. pneumoniae* species used as references. Log scores were calculated based on comparisons between the unidentified spectra and the database’s reference spectra. Identification at the species level was reliable when scores equaled or exceeded 2.0, while identification at the genus level was reliable when scores ranged between 1.9 and 1.7. The Main Spectrum Profile (MSP) dendrogram was created to measure the correlation distance among MBT spectra for clustering. Distances of 500, 180, and 100 were used to separate and examine clusters. As a positive control, Escherichia coli was used as the bacterial test standard throughout the experiment.

### Molecular identification of *K. pneumoniae* using SYBR green real-time PCR

#### DNA extraction

The DNA was extracted using a thermal lysis technique (Ahmed et al. 2013). In brief, 3–5 bacterial colonies were added to 200 µL of distilled water in a PCR tube. The PCR tube was then heated at 98 °C for 20 min using a thermal cycler (Biometra, Göttingen, Germany). Afterward, cellular debris was pelletized by centrifuging the tubes for 5 min at 14.000 rpm. We subsequently transferred the supernatant into sterile, labeled Eppendorf tubes and stored them at -20 °C for further processing.

#### Current investigation’s primers

The isolates were further confirmed for the presence of virulence-associated *K. pneumoniae* genes by analyzing their *16 S rRNA*, microviscosity-associated A (*magA*), allantoin metabolism (*allS*), ferric iron uptake (Kfu), capsule-associated (*K2A*), regulator of mucoid phenotype A (*rmpA*), and enterobactin gene (*entB*). For the detection of *K. pneumoniae*, a specific region of the *16 S rRNA* was selected carefully and the primers were selected using the Primer Express software, version 2.0 (Applied Biosystems, USA), and tested for specificity using the Basic Local Alignment Search Tool (BLAST) (Table 1).

**Table 1:** Primer sets for detecting virulence-associated genes in *K. pneumoniae*

Gene target	DNA sequence (5’ to 3’)	Base pair (bp)	References
*16 S rRNA*	ATT TGA AGA GGT TGC AAA CGA T	130	Song et al. (2003)
	TTC ACT CTG AAG TTT TCT TGT GTT C		
*magA*	GGT GCT CTT TAC ATC ATT GC	1283	Turton et al. (2010)
	GCA ATG GCC ATT TGC GTT TGC GTT AG		
*allS*	CATTACGCACCTTTGTCAGC	764	Compain et al. (2014)
	GAATGTGTCGGCGATCAGCTT		
*Kfu*	GGCCTTTGTCCAGAGCTACG	638	Compain et al. (2014)
	GGGTCTGGCGCAGAGTATGC		
*k2A*	CAACCATGGTGGTCGATTAG	543	Struve et al. (2005)
	TGGTAGCCATATCCCTTTGG		
*rmpA*	ACT GGG CTA CCT CTG CTT CA	536	Yeh et al. (2006)
	CTT GCA TGA GCC ATC TTT CA		
*entB*	GTCAACTGGGCCTTTGAGCCGTC	400	Compain et al. (2014)
	TATGGGCGTAAACGCCGGTGAT		

#### Protocol of SYBR green real-time PCR assay and electrophoresis of PCR products

An Applied Biosystems 7500 Fast Real-Time PCR System was then used for the detection of the virulence-associated genes of *K. pneumoniae*. The reaction volume for this experiment was 10 µL, which consisted of 5 µL of SYBR Master mix (2x) (MedChemistryExpress, Monmouth Junction, USA), 0.2 µL of forward primer (10 µM), 0.2 µL of reverse primer (10 µM), 1 µL of DNA, and 3.4 µL of RNase/DNase free water (ddH_2_O). The following amplification parameters were used: 50 °C for 2 min, 95 °C for 2 min, followed by 40 amplification cycles consisting of 15 s at 95 °C and 1 min at 60 °C. Water was used as the negative control rather than template DNA for the PCR reaction. To ensure precise findings, each reaction was performed twice. A plot of Delta Rn (Rn) versus cycle number was used to identify the *K. pneumoniae* genes from the qPCR results. A LabChip GX Touch 24 system (PerkinElmer, Massachusetts, United States) was then applied for electrophoresis of the PCR products. According to the manufacturer’s instructions, DNA 1 K Assay Quick was used to prepare and chip the samples.

#### Antibiotic susceptibility testing using Vitek 2 compact AST cards

The Vitek2® Gram Negative Susceptibility cards (AST-GN66 Test Kit 20 Cards − 413,398) purchased from Biomérieux, Lyon, France were used to assess antibiotic resistance and susceptibility against identified strains of *K. pneumoniae* according to minimal inhibitory concentrations (MICs). Based on the manufacturer’s guidelines, the isolate suspension was prepared and then adjusted using a Vitek Densichek, which measures the optical density of a microbe suspension (0.5 to 0.63). A summary of the MIC ranges (µg/mL) of the 17 antimicrobial agents of 10 various classes of antibiotics included on the AST-GN66 card is presented in Table 4. Based on the instructions provided by the manufacturer, AST-GN66 cards were inoculated in bacterial test tubes and then submitted to the device for interpretation. The MIC was classified as susceptible, moderate, or resistant based on the permissions granted by the Clinical and Laboratory Standards Institute (CLSI) (Cockerill et al. 2013). According to the CLSI (Clinical and Laboratory Standards Institute 2014), the term “multidrug-resistant” (MDR) strain is used to describe bacteria that are resistant to at least one antibiotic across three or more chemical families. The term “extensively drug-resistant” (XDR) strain describes bacterial isolates that are resistant to at least one antibiotic agent in all but one or two antimicrobial subcategories. The term “pan drug-resistant” (PDR) refers to bacteria that are non-susceptible to all antimicrobial agents. *K. pneumoniae* Deutsche Sammlung von Mikroorganismen und Zellkulturen (DSMZ) 26,371 was used as a positive control strain.

#### Phynotypic detection of extended-spectrum β-lactamases (ESBL) in *K. pneumoniae* isolates

This study utilized the double disc synergy test (DDST) to identify ESBL *K. pneumoniae*. In brief, discs of amoxicillin/clavulanic acid (20 g/10 g) and third-generation cephalosporin discs (30 g ceftazidime, 30 g cefotaxime, and 30 g cefpodoxime) were placed on a Muller-Hinton agar plate (Arkan, Burayidah, Saudi Arabia) approximately 20 mm apart from each other (Duttaroy and Mehta 2005). Each plate was incubated overnight at 37 °C. The presence of an increase in the cephalosporin inhibition zone toward the amoxicillin/clavulanic acid disc was considered a positive indicator of ESBL. We used *K. pneumoniae* DSM 681 as a control.

### Statistical analysis

As soon as the findings of the investigation were received, all calculations were performed using SPSS, version 20.0 (SPSS Inc., Chicago, IL, USA).

## Results

A total of 190 isolates were obtained from several hospitals in Al-Qassim, Saudi Arabia, between March 2021 and October 2022. They were categorized into 69 urine isolates, 52 respiratory isolates, 48 wound isolates, and 21 blood isolates. The Vitek® 2 ID Compact system, Gram staining, and culture were the primary methods employed for identifying *K. pneumoniae* isolates. An automated MALDI Biotyper (MBT) device was utilized for proteome identification. SYBR green real-time polymerase chain reaction (real-time PCR) and microfluidic electrophoresis were employed to confirm these findings. Additionally, we screened the isolates for antibiotic susceptibility and resistance.

### Phenotypic characteristics of the recovered *K. pneumoniae* isolates

A total of 190 *K. pneumoniae* isolates were preliminarily identified by culture and staining techniques from blood, respiratory, and wound samples collected from three hospitals in the Al-Qassim region of Saudi Arabia. As shown in Table 2 and 178 (93.68%) isolates were correctly identified by the Vitek 2 Compact ID system, of which 95.65%, 93.30%, and 93.75% were obtained from urinary, respiratory, wound, and blood samples, respectively. Ten (6.32%) isolates were misidentified, of which 3 (4.35%) were recovered from urine, 4 (6.70%) from respiratory, and 3 (6.25%) from wounds. On the other hand, two (9.53%) of the isolates isolated from blood were not identified.

**Table 2:** The distribution of *K. pneumoniae* isolates from various clinical samples identified by the Vitek 2 compact system

Source of isolation	Number of isolates	Correctly identified		Misidentified		Not identified	
		Number	Percentage	Number	Percentage	Number	Percentage
Urinary	69	66	95.65	3	4.35	0	0.00
Respiratory	52	48	93.30	4	6.70	0	0.00
Wound	48	45	93.75	3	6.25	0	0.00
Blood	21	19	90.47	0	0.00	2	9.53
Total	190	178	93.68	10	6.32	0	0.00

### Proteomic identification of *K. pneumoniae* using PMF technique

MBT automated equipment was used to analyze 190 isolates of *K. pneumoniae*. Using the Bruker library of Compass software to compare the obtained spectra, 188/190 (98.95%) were successfully recognized. At the species level, 103 isolates (54.21%) were identified with scores ranging from 2.30 to 3.00, and 85 isolates (44.54%) were identified with scores ranging from 2.00 to 2.29. Two isolates could be identified at the genus level, with score values ranging from 1.7 to 1.99. The remaining two isolates were not identified.

**Fig. 1:**
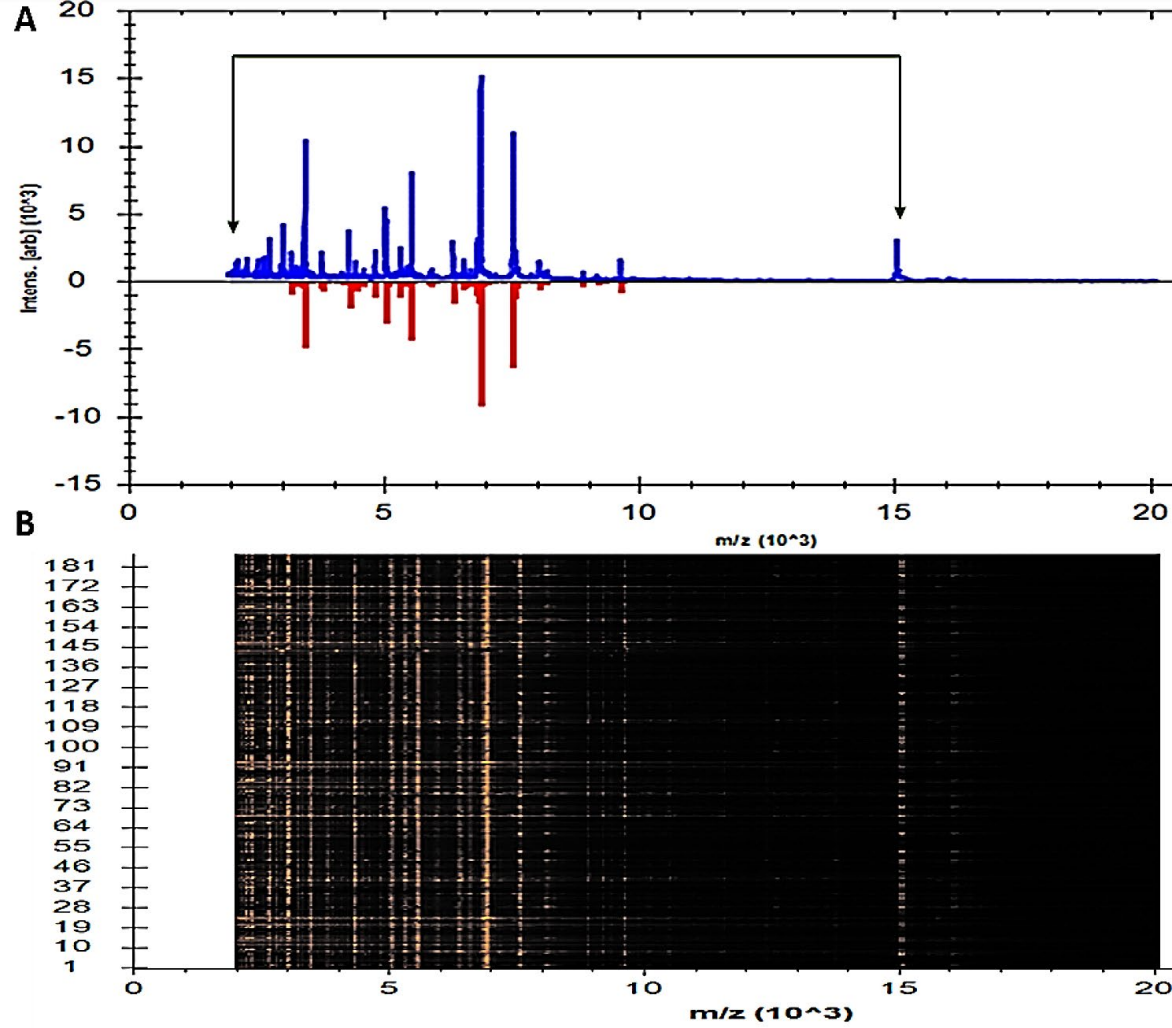
Protein profiles of 190 *K. pneumoniae*; **A** peak distributions within line spectra between 2150 and 15,043 m/z; **B** gel profiles from different protein spectra accumulated together

**Fig. 2:**
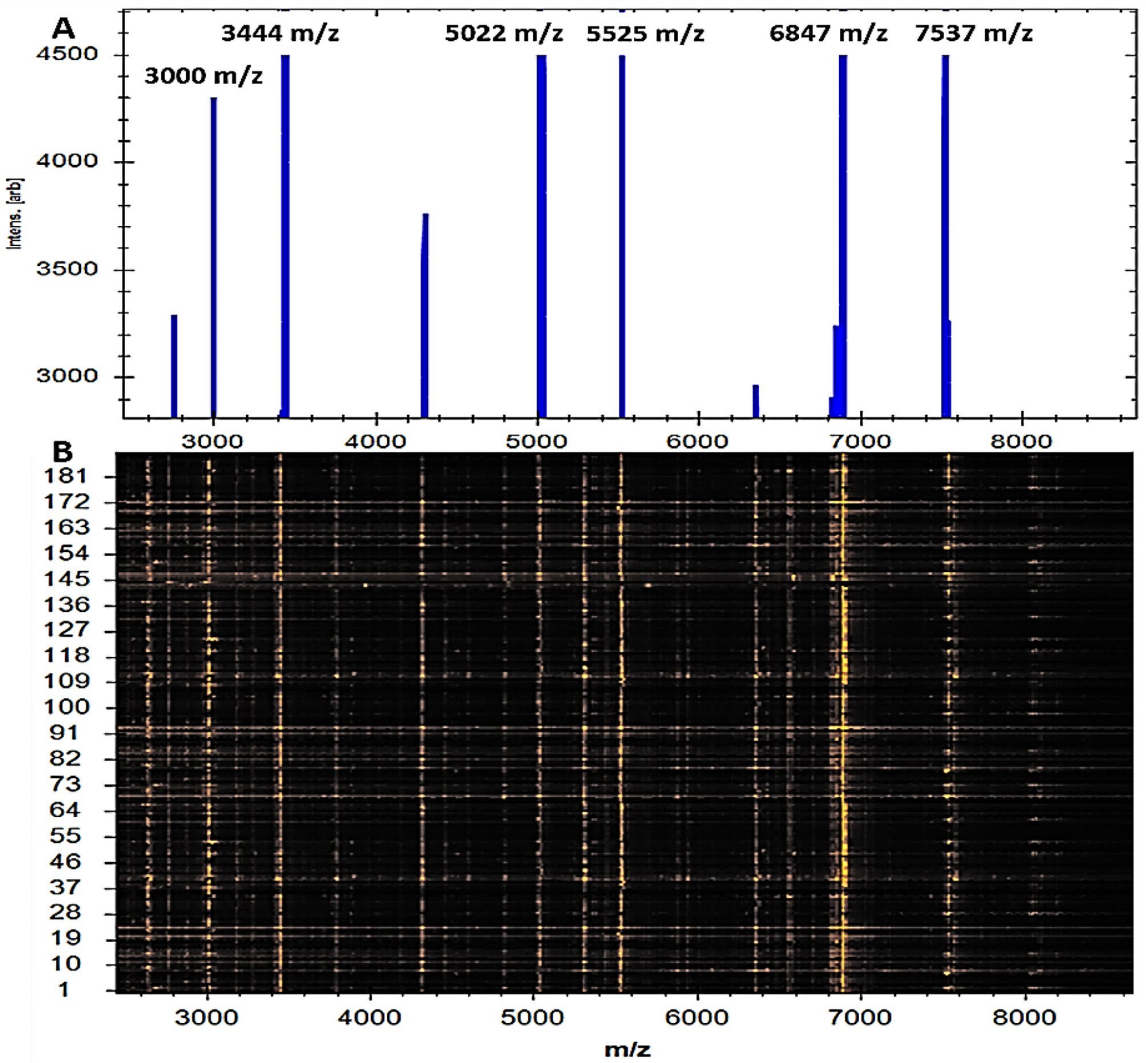
Mass spectroscopic patterns of 190 *K. pneumoniae*; **A** strong peaks were seen at 3444, 5022, 5525, 6847, and 7537 m/z; and **B** the gel pattern reflected protein spectra scattered throughout the same region

**Fig. 3:**
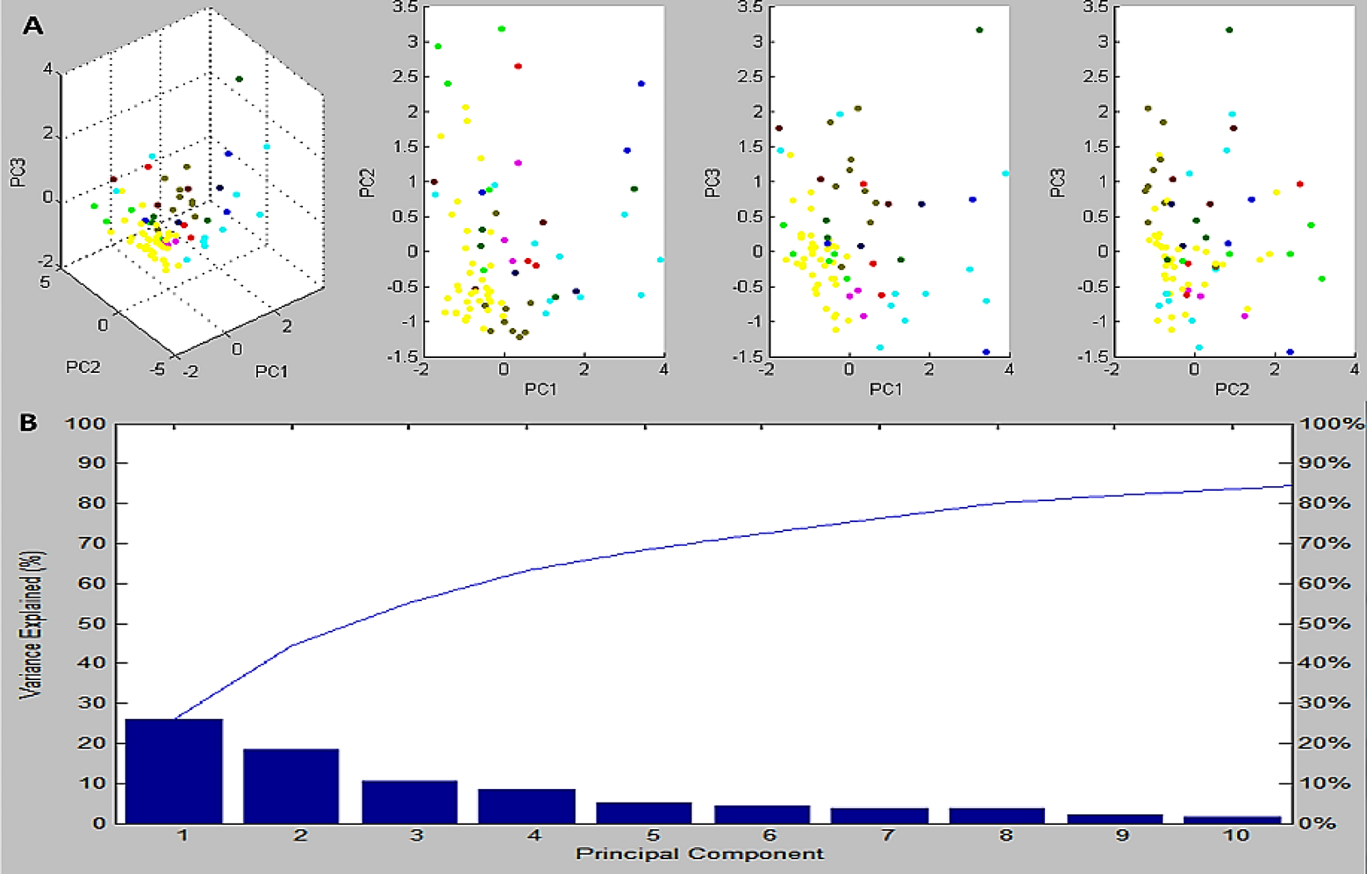
Analysis of the peak lists of *K. pneumoniae* isolates analyzed using the Peptide Mass Fingerprinting (PMF) technique. **A** the distribution of *K. pneumoniae* isolates isolated from urine (yellow), respiratory (cyan), wound (olive), and blood (pink) samples can be observed in the diagram illustrating the first three principal components (PCs) of the entire peak lists of PMF mass spectra, and **B** the contribution of ten PC models to the classification of profiles is shown in the plot indicating the proportion of variance explained by the PC model

As can be seen in a recent gel image, each *K. pneumoniae* isolate produced distinct spectra. In Fig. 1, numerous spectra were observed between 2150 and 15,043 m/z, whereas in Fig. 2, the highest peak values were found in 3444, 5022, 5525, 6847, and 7537 m/z. Our study also utilized a complementary computational technique called Principal Component Analysis (PCA) derived from the MBT Compass software in order to investigate protein spectra variance and consistency. It can be seen in Fig. 3a that in 3D PCA, there were a number of distinct spectral patterns for each protein of the detected isolates. For the purpose of identifying each peak, three loading values were used. The loading values for these components were derived from the three principal components (PC1, PC2, and PC3) equation. In our investigation, all reported peaks in the MBT library were examined with the help of the PCA tool. As can be seen in the 3D PCA, all *K. pneumoniae* isolates were grouped together into one group. It was estimated by PCA that PC1, PC2, and PC3 had reciprocal effects of approximately 45%, 17%, and 9%, respectively, on the development of a profile in a percentage plot of the difference clarified (Fig. 3b).

**Fig. 4:**
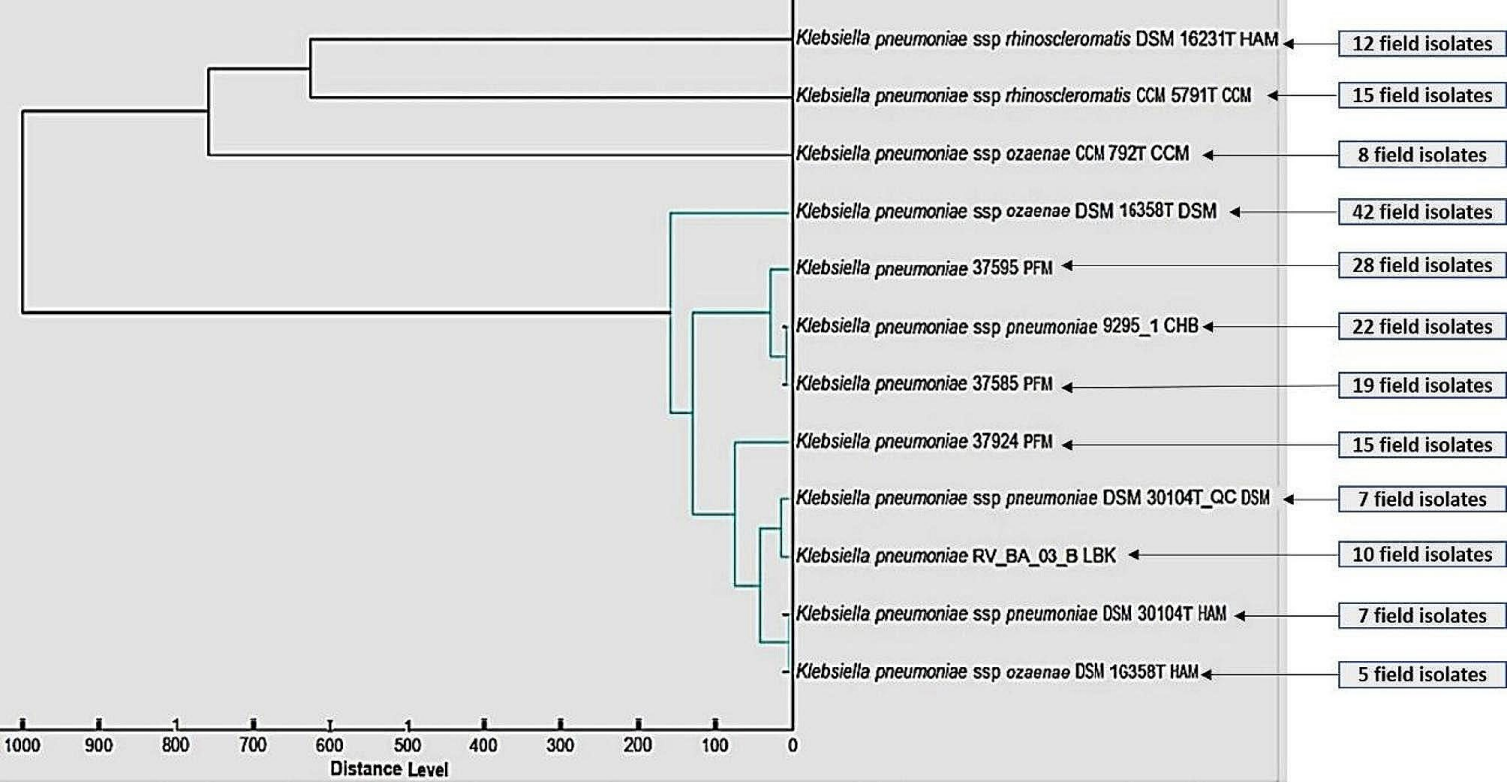
Bruker’s library contains 12 reference strains that were matched with 190 clinical isolates of *K. pneumoniae*. In this diagram, the numbers of the field-isolated strains are shown in front of the corresponding reference strain and marked with arrows

Based on our findings, the MSP dendrogram revealed that the evaluated *K. pneumoniae* strains exhibited strict associations with twelve reference strains stored in the Bruker library (Fig. 4). In the analysis of the results, 190 clinical isolates of *K. pneumoniae* were matched with the following reference strains: forty-two isolates with *K. pneumoniae* ssp. ozaenae DSM 16358T DSM, twenty-eight isolates with *K. pneumoniae* 37,595 PFM, twenty-two isolates with *K. pneumoniae* ssp. *pneumoniae* 9295_1 CHB, nineteen isolates with *K. pneumoniae* 37,585 PFM, and fifteen isolates with *K. pneumoniae* 37,924 PFM. Furthermore, fifteen strains matched *K. pneumoniae* ssp. *ozaenae* CCM 5791T CCM, twelve strains matched *K. pneumoniae* ssp. *rhinoscelromatis* DSM 16231T HAM, 10 strains matched *K. pneumoniae* RV_BA_03_B LBK, 8 strains matched *K. pneumoniae* ssp. ozaenae CCM 792T CCM 7 strains matched *K. pneumoniae* ssp. *pneumoniae* DSM 30104T_QC DSM, 7 strains matched *K. pneumoniae* ssp. *pneumoniae* DSM 30104T HAM and 5 isolates matched *K.pneumoniae* ssp. *ozaenae* DSM 1G358T HAM. Matching field isolates with reference strains in the Bruker library using MALDI is a method used to identify unknown bacteria. This is done by comparing the mass spectrum of the unknown bacteria to that of a known bacterium in the database. If the spectra match, the unknown bacteria can be confidently identified.

### Verification of the identification of *K. pneumoniae* isolates with SYBR green real-time PCR

Following the MBT assay, the results were validated using SYBR Green Real-Time PCR. A group of primers was developed specifically targeting sections of the *16 S rRNA*, *magA*, *allS*, *Kfu*, *k2A*, *rmpA*, and *entB* genes to distinguish virulent strains of *K. pneumoniae*. Amplification of these primers resulted in amplified fragments with the specified molecular weights during PCR amplification. Separately, the amplification of each gene was evaluated, and the size of each anticipated product was determined. After LabChip analysis, the PCR product sizes for *16 S rRNA*, *magA*, *allS*, *Kfu*, *k2A*, *rmpA*, and *entB* were 130 bp, 1283 bp, 764 bp, 638 bp, 543 bp, 536 bp, and 400 bp, respectively. As can be seen in Tables 4 and 172 (90.53%), 133 (70%), 62 (32.63%), 23 (12.11%), 7 (3.63%), 3 (1.58%), and 1 (0.53%) of the isolates carried the *entB*, *16 S rRNA*, *kfu*, *rmpA*, *k2A*, *allS*, and *magA* genes, respectively. A total of 175 (92.11%) *K. pneumoniae* isolates were positive for numerous virulence genes, but only one strain contained the *magA* gene. It has been demonstrated that the results obtained by MBT and SYBR Green Real-time-PCR were in perfect agreement; therefore, the results obtained by the PCR confirmed those obtained by MBT.

**Table 3:** The prevalence of *K. pneumoniae* virulence associated genes in various clinical specimens

Source of isolation	Number of isolates	16 S rRNA		magA		allS		Kfu		k2A		rmpA		entB	
		No.	%	No.	%	No.	%	No.	%	No.	%	No.	%	No.	%
Urinary	69	47	68.12	1	1.45	1	1.45	19	27.54	3	4.35	7	10.14	61	88.40
Respiratory	52	37	71.15	0	0.00	2	2.90	17	32.69	1	1.92	5	9.62	49	94.23
Wound	48	33	68.75	0	0.00	0	0.00	20	41.67	2	4.17	5	10.42	45	93.75
Blood	21	16	76.19	0	0.00	0	0.00	6	28.57	1	4.76	3	14.29	17	80.96
Total	190	133	70.00	1	0.53	3	1.58	62	32.63	7	3.68	23	12.11	172	90.53

### Antibiotic resistance of *K. pneumoniae*

**Fig. 5:**
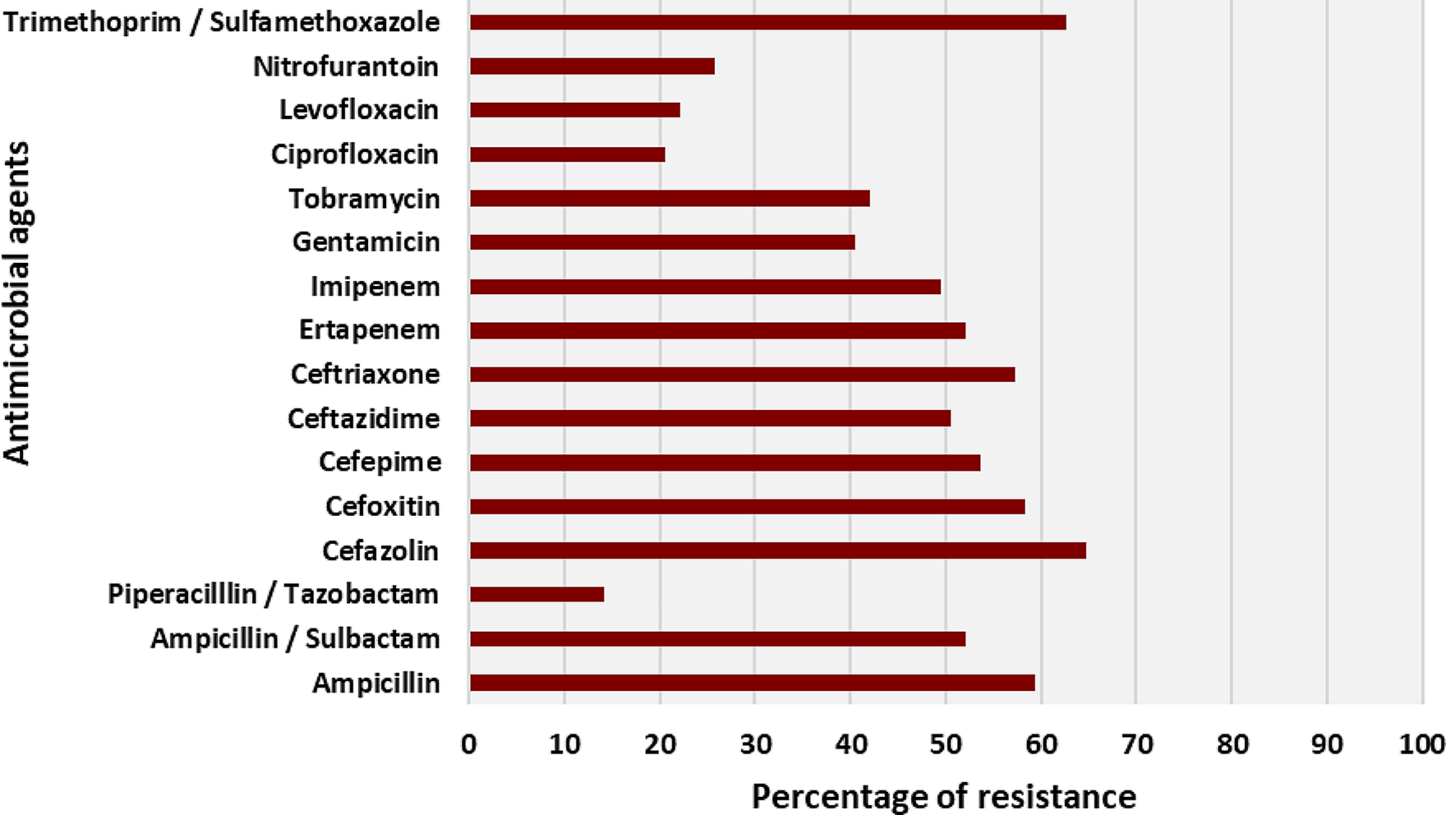
Proportions of *K. pneumoniae* isolates resistant to various antimicrobial agents

As shown in Fig. 5; Table 4, among 190 *K. pneumoniae* isolates, 123 (64.75%) were resistant to cefazolin (cephalosporin I), 119 (62.63%) to trimethoprim/sulfamethoxazole (sulfonamides), 113 (59.45%) and 99 (52.11%) were resistant to ampicillin/ampicillin-sulbactam (aminopenicillin/inhibitor combination), and 111 (58.42%) were resistant to cefoxitin (cephalosporin II/cephamycin). Moreover, 109 (57.37%), 102 (53.68%), 96 (50.53%), 99 (52.11%), and 94 (49.47%) isolates were resistant to ceftriaxone, cefepime, ceftazidime (cephalosporin III/IV), and ertapenem/Imipenem (carbapenem), respectively. A variable degree of resistance was also observed for the class of aminoglycosides, including tobramycin (42.11%) and gentamicin (40.53%). In contrast, the isolates of *K. pneumoniae* were highly susceptible to ureidopenicillin/inhibitor combinations, including piperacillin/tazobactam (87.79%), fluoroquinolones, including levofloxacin (77.89%), and nitrofurans including nitrofurantoin (74.21%) (Table 4). Based on the results of the DDST, 93/190 (48.95%) isolates of *K. pneumoniae* were ESBL, while the remainder were non-ESBL.

**Table 4:** Using Vitek2® Compact AST-GN66 cards to determine the antibiotic susceptibility and resistance percentages of 190 *K. pneumoniae* isolates obtained from clinical samples

Class of antibiotic	Antibiotic used	MIC range (µg/ml)	Results of AST-GN66 cards					
			Susceptible		Intermediate		Resistant	
			No.	%	No.	%	No.	%
Aminopenicillin/inhibitor combination	Ampicillin	2–32	77	40.55	0	0.00	113	59.45
	Ampicillin/Sulbactam	2/1–32/16	91	47.89	0	0.00	99	52.11
Ureidopenicillin/inhibitor combinations	Piperacilllin/Tazobactam	4/4–128/4	163	85.79	0	0.00	27	14.21
Cephalosporin I	Cefazolin	4–64	43	22.62	24	12.63	123	64.75
Cephalosporin II/Cephamycin	Cefoxitin	4–64	79	41.58	0	0.00	111	58.42
Cephalosporin III/IV	Cefepime	1–64	88	46.32	0	0.00	102	53.68
	Ceftazidime	1–64	94	49.47	0	0.00	96	50.53
	Ceftriaxone	1–64	71	42.63	0	0.00	109	57.37
Carbapenem	Ertapenem	0.5–8	91	47.89	0	0.00	99	52.11
	Imipenem	0.25–16	96	50.53	0	0.00	94	49.47
Aminoglycosides	Gentamicin	1–16	110	57.89	3	1.58	77	40.53
	Tobramycin	1–16	110	57.89	0	0.00	80	42.11
Fluroquinolones	Ciprofloxacin	0.25–4	139	73.15	12	6.32	39	20.53
	Levofloxacin	0.12–8	148	77.89	0	0.00	42	22.11
Nitrofurans	Nitrofurantoin	16–512	141	74.21	0	0.00	49	25.79
Sulfonamides	Trimethoprim/Sulfamethoxazole	20 (1/19)–320 (16/304)	66	34.74	5	2.63	119	62.63

## Discussion

In accordance with recent data from the Centers for Disease Control and Prevention, two-thirds of all infections linked to hospital settings are caused by bacteria belonging to the ESKAPE group, which significantly contributes to global mortality and morbidity (Córdova-Espinoza et al. 2023). Middle Eastern healthcare-associated infections (HAIs) are predominantly bloodstream and surgical wound infections, followed by urinary tract infections (Nimer 2022). Gram-negative bacteria, including *Escherichia coli* and *Klebsiella* species, are the most commonly identified pathogens involved in HAIs (Nimer 2022). Worldwide, *K. pneumoniae* is responsible for more than 600,000 deaths related to antibiotic resistance, making it second only to *E. coli* in terms of prevalence (Al Bshabshe et al. 2020; Hafiz et al. 2023). Analytical methods that can be used in real-time and with high precision are necessary to monitor HAIs and screen for any undesired pathogens that may pose serious risks to patient health. Keeping *K. pneumoniae* at bay is essential due to its association with a variety of human illnesses, and so correctly identifying it is a crucial part of diagnosis (Russo et al. 2018; Nyblom et al. 2023; Zhang et al. 2023). A fast turnaround time is also essential in order to begin treatment as soon as possible (Bassetti et al. 2018; Boattini et al. 2023). Due to its quick, precise, and inexpensive nature, PMF has become an essential tool for the early detection of bacteria in clinical and environmental samples over the past two decades (Elbehiry et al. 2021, 2022, 2023; Alzaben et al. 2022).

According to our study, 188 out of 190 isolates of *K. pneumoniae* recovered from various clinical samples were correctly identified (98.95%). Based on these findings, the mass spectrum provided by MBT Compass 2.0 Software was sufficient to identify all isolates of *Klebsiella* at the species level. We upgraded Compass 2.0 for use in our investigation, which may explain the improved accuracy compared to previous investigations. Similar results were reported by Bridel et al. (Bridel et al. 2021), who developed a web-based tool called *Klebsiella* MALDI TypeR to identify environmental and clinical isolates of *Klebsiella*. They reported that this method can quickly produce results based on batches of uploaded spectral data, with an accuracy ranging between 84% and 100% depending on the phylogroup. According to Kumar et al. (Kumar et al. 2020), MALDI-TOF MS correctly identified 20 *K. pneumoniae* (100%) isolates at the species level with scores equal to or greater than 2.0. Another study by Roncarati et al. (Roncarati et al. 2021) used cluster analysis of MALDI-TOF MS-created spectra to identify 65 *K. pneumoniae* isolates from positive blood cultures. Based on their findings, 62 out of 65 (95.4%) of the *K. pneumoniae* strains were correctly identified. Furthermore, Cuello and his colleagues (Cuello et al. 2023) used MALDI-TOF Mass Spectrometry Technology to identify *K. pneumoniae* isolates from clinical samples and found that this technology had 100% specificity. Having a reliable database and obtaining high-quality spectra are essential for reliable identification (Cuénod et al. 2023). One of the commercial databases used in routine diagnostics is the MBT Compass reference library version 4.1.100. This library includes more than fifteen *K. pneumoniae* reference strains, increasing the likelihood of accurate identification.

Like any other microbiological detection method, MALDI-TOF MS has both advantages and disadvantages (Elbehiry et al. 2021; Elbehiry, 2022; Haider et al. 2023). This technique can detect a variety of microorganisms, including Gram-positive and Gram-negative bacteria, as well as fungi (Biswas and Rolain 2013). However, spectral disruption may occur due to the presence of endospores from bacteria like Bacillus species. To avoid this issue, it is recommended to use 24-hour cultures (Clark et al. 2013). MALDI-TOF MS is effective in identifying species at the species level for anaerobic isolates from clinical samples, but it is less likely to identify Gram-positive isolates. Therefore, it is suggested that reference spectra from this bacterial group be included in future reference database releases (Chalupová et al. 2014). MALDI-TOF has been used to analyze various clinical samples, such as milk, urine, and blood, and has proven to be precise in identifying bacteria (Croxatto et al. 2016; Wilson et al. 2019). However, the presence of a large number of germs in blood and urine samples hinders the widespread application of this method. To overcome this obstacle, various methods, such as urine filtering and blood separation, are available (Tsuchida et al. 2020).

SYBR Green Real-Time PCR was successfully used in this study to confirm the detection of *K. pneumoniae* in different clinical samples. There was a strong correlation between the PMF approach and PCR analysis for the *16 S rRNA*, *magA*, *allS*, *Kfu*, *k2A*, *rmpA*, and *entB* genes, regardless of the source of the isolates (urine, wound, blood, and exudate). The PCR product can be demonstrated within five minutes using a microchannel fluidic device, whereas LabChip requires twenty minutes for preparation. By combining protein analysis with molecular methods, unconfirmed *K. pneumoniae* isolates could be identified in approximately 2.5 h. The pathogenicity of *K. pneumoniae* can be attributed to various factors that contribute to its virulence, including the capsule, endotoxins, siderophores, iron-scavenging systems, and adhesins. These elements enable the bacterium to evade the host’s immune system and cause different types of illnesses (Ahmed and Alaa 2016; Zhang et al. 2018; Remya et al. 2019).

One hundred and seventy-five out of 190 isolates examined in the current investigation were found to possess 92.11% of multiple genes coding for virulence. Almost all *K. pneumoniae* strains carried at least one siderophore. Among the 190 isolates, 90.53% (172/190) expressed the *entB gene*. The respiratory isolates demonstrated the highest expression of this gene (94.23%), followed by the wound isolates (93.75%), the urine isolates (88.40%), and the blood isolates (80.96%). Previous studies have identified *entB* as the most frequent siderophore found in *K. pneumoniae* (El Fertas-Aissani et al. 2013; Ahmed and Alaa 2016; Remya et al. 2019; Chen et al. 2020; Han et al. 2022). Another gene, known as *Kfu*, encodes an iron absorption mechanism that plays a role in invasion and capsule production [3,30]. According to our investigation, 62 out of 190 isolates (32.63%) expressed this gene. Among the isolates positive for this gene, 41.67% were from wounds, followed by respiratory secretions (32.69%), blood (28.57%), and urine (27.54%). Our findings align with those of a study conducted in Iraq (Ahmed and Alaa 2016). Previous studies have also found this gene in isolates of *K. pneumoniae* (LiuGuo 2019; Tan et al. 2019).

A total of 23 isolates (12.11%) tested positive for the *rmpA* gene, as shown in Table 3. The presence of this gene may indicate a high virulence potential in an isolate. According to a previous investigation, the *rmpA* gene is embedded in a 180-kb virulence plasmid. This multicopy plasmid induces *K. pneumoniae* to express a mucoid phenotype (Walker et al. 2019). The *rmpA*-carrying plasmids of *K. pneumoniae* isolates have also been found to contain several other virulence-associated genes (Wang et al. 2020). In a study by Yu et al. (2006), 72 out of 151 isolates contained the *rmpA* gene. When examining the frequency of the *rmpA* gene, Yu et al. (Yu et al. 2006) found several virulence factors in 96% of the causative isolates. Additionally, liver abscess isolates with a positive hyper-mucoviscosity phenotype had an average frequency of 97.8% for the *rmpA* gene. According to our findings, *K. pneumoniae* contains genes associated with its virulence, which makes it a virulent bacterium. Even having just one virulence gene of *K. pneumoniae* can result in adverse consequences. Among the genes detected in the current investigation, *EntB, 16 S rRNA*, and *Kfu* are the most frequently associated with the emergence of various illnesses.

In this study, we investigated the patterns of antimicrobial resistance among *K. pneumoniae* isolates from various clinical samples. Previous studies have indicated that this bacterium is becoming one of the most multidrug-resistant pathogens worldwide (Cepas et al. 2019; Sakkas et al. 2019; Ibrahim 2023). We assessed the susceptibility and resistance of 190 *K. pneumoniae* isolates to antimicrobial drugs commonly prescribed for gram-negative bacteria using the Vitek 2 Compact AST cards. Our results indicate that certain isolates of *K. pneumoniae* were resistant to various classes of antibiotics such as cephalosporin I [cefazolin (64.75%)], cephalosporin II/cephamycin [cefoxitin (58.42%)], cephalosporin III/IV [cefepime (53.68%), ceftazidime (50.53%), and ceftriaxone (57.37%)], sulfonamides [trimethoprim/sulfamethoxazole (62.63%)], carbapenems [ertapenem (52.11%) and imipenem (49.47)], and aminoglycosides [gentamicin (40.53%) and tobramycin (42.11%)]. Similar results have been obtained by other research studies conducted around the world (Azim et al. 2019; Badger-Emeka et al. 2021; Lagha et al. 2021).

In an additional study, Audi and Naeem (Audi and Naeem 2023) examined sixty *K. pneumoniae* isolates taken from 300 urine samples and determined their resistance to various antibiotics. According to their findings, all isolates were resistant to ampicillin (100%), cefazolin (53.3%), imipenem (5%), and tigecycline (3.3%), respectively. Among the multidrug-resistant *K. pneumoniae* strains isolated from Bangladesh, Aminul et al. (Aminul et al. 2021) discovered that the strains developed high levels of resistance to cotrimoxazole (86.9%), ciprofloxacin (80.4%), entamicin (72.3%), ceftriaxone (89.4%), ceftazidime (87.8%), and piperacillin-tazobactam (51.2%). A study conducted in Pakistan by Ullah et al. (Ullah et al. 2009) displayed similar results for cotrimoxazole (93.48%) and gentamicin (52.17%). Nevertheless, ceftriaxone, ceftazidime, amikacin, ciprofloxacin, meropenem, and piperacillin-tazobactam revealed different levels of resistance (54.35%, 54.35%, 32.61%, 52.1%, 6.52%, and 39.13%, respectively).

The ESKAPE group, particularly *K. pneumoniae* and *E. coli* in healthcare-associated infections (HAIs), continuously develop new defense mechanisms known as “resistance mechanisms” to evade antimicrobial agents. They do this by producing enzymes called extended-spectrum beta-lactamases (ESBLs) (Jiang et al. 2018; Ayobami et al. 2022; Alcántar-Curiel et al. 2023). These enzymes break down and destroy commonly used antimicrobial agents like penicillins and cephalosporins, rendering them ineffective. In brief, there are five key mechanisms by which *K. pneumoniae* is resistant to antibiotics, including enzymatic inactivation and modification of antibiotics, alteration of antibiotic targets, loss and mutation of porins, enhanced efflux pump expression, and biofilm development (Sikarwar and Batra 2011; Mulani et al. 2019).

In the current investigation, 93 out of 190 (48.95%) *K. pneumoniae* isolates were found to be ESBL producers. This rate is similar to a previous study conducted in Bangladesh by Chakraborty et al. (Chakraborty et al. 2016), where 45% of *K. pneumoniae* produced ESBL. Another study conducted in central Côte d’Ivoire showed an even higher percentage of ESBL-producing *K. pneumoniae* isolates (84%) collected from various clinical samples (Müller-Schulte et al. 2020). The increasing antibiotic resistance has limited the available treatment options for infections caused by ESBL-producing Enterobacterales (Padmini et al. 2017; Tamma et al. 2021). Most diseases caused by ESBL-producing *K. pneumoniae*, such as urinary tract infections, require more advanced therapies (Bitsori and Galanakis 2019). Among the few antibiotics effective against ESBL-producing bacteria, carbapenems (such as ertapenem and imipenem) are highly effective. However, there is also an increase in resistance enzymes that degrade these drugs (Tilahun et al. 2021).

In conclusion, Peptide mass fingerprinting (PMF) is a powerful analytical technique used to identify *K. pneumoniae* isolates from various clinical samples based on their proteomic characteristics. To confirm the findings of PMF, qPCR and microchannel fluidics electrophoresis assays were employed to detect virulence-associated genes in *K. pneumoniae* isolates. This technology has the potential to be used in the future for direct identification and differentiation of *K. pneumoniae* in environmental and hospital samples. The enterobactin siderophores (*entB*) gene was found to be the most frequently detected gene in *K. pneumoniae* isolates. Additionally, *K. pneumoniae* isolates have displayed increasing resistance to antibiotics from different classes, including carbapenem. This poses a significant threat to human health, as infections may become difficult to treat.

## Data Availability

All data produced or analysed during this study are included in this published article.
